# Long term study on the effect of mollusciciding with niclosamide in stream habitats on the transmission of schistosomiasis mansoni after community-based chemotherapy in Makueni District, Kenya

**DOI:** 10.1186/1756-3305-6-107

**Published:** 2013-04-18

**Authors:** Henry C Kariuki, Henry Madsen, John H Ouma, Anthony E Butterworth, David W Dunne, Mark Booth, Gachuhi Kimani, Joseph K Mwatha, Eric Muchiri, Birgitte J Vennervald

**Affiliations:** 1 Division of Vector Borne Diseases, Kenya Ministry of Health, Nairobi, Kenya; 2 Present address: Kenya Methodist University, School of Medicine and Health Sciences, P.O. Box 267, Meru, Kenya; 3 DBL Centre for Health Research and Development, Institute of Veterinary Disease Biology, University of Copenhagen, Copenhagen, Denmark; 4 Maseno University, Kisumu, Kenya & c/o PO Box 57864, Nairobi, Kenya; 5 College of Medicine, University of Malawi, Blantyre, Malawi; 6 Department of Pathology, University of Cambridge, Cambridge, UK; 7 Present address: School of Medicine, Pharmacy and Health, Durham University, Durham, UK; 8 Kenya Medical Research Institute, Nairobi, Kenya

**Keywords:** Bayluscide, *Schistosoma mansoni*, Re-infection, *Biomphalaria pfeifferi*, Molluscicide

## Abstract

**Background:**

*Schistosoma mansoni* infection is a persistent public health problem in many Kenyan communities. Although praziquantel is available, re-infection after chemotherapy treatment is inevitable, especially among children. Chemotherapy followed by intermittent mollusciciding of habitats of *Biomphalaria pfeifferi*, the intermediate host snail, may have longer term benefits, especially if timed to coincide with natural fluctuations in snail populations.

**Methods:**

In this cohort study, the Kambu River (Intervention area) was molluscicided intermittently for 4 years, after mass chemotherapy with praziquantel in the adjacent community of Darajani in January 1997. The nearby Thange River was selected as a control (Non-intervention area), and its adjacent community of Ulilinzi was treated with praziquantel in December 1996. Snail numbers were recorded monthly at 9–10 sites along each river, while rainfall data were collected monthly, and annual parasitological surveys were undertaken in each village. The mollusciciding protocol was adapted to local conditions, and simplified to improve prospects for widespread application.

**Results:**

After the initial reduction in prevalence attributable to chemotherapy, there was a gradual increase in the prevalence and intensity of infection in the non-intervention area, and significantly lower levels of re-infection amongst inhabitants of the intervention area. Incidence ratio between areas adjusted for age and gender at the first follow-up survey, 5 weeks after treatment in the non-intervention area and 4 months after treatment in the intervention area was not significant (few people turned positive), while during the following 4 annual surveys these ratios were 0.58 (0.39-0.85), 0.33 (0.18-0.60), 0.14 (0.09-0.21) and 0.45 (0.26-0.75), respectively. Snail numbers were consistently low in the intervention area as a result of the mollusciciding. Following termination of the mollusciciding at the end of 2000, snail populations and infections in snails increased again in the intervention area.

**Conclusion:**

The results of this study demonstrate that in the Kenyan setting a combination of chemotherapy followed by intermittent mollusciciding can have longer term benefits than chemotherapy alone.

## Background

Chemotherapy with praziquantel, directed at *Schistosoma mansoni* infected primary school children, is an effective way of causing a rapid reduction in morbidity and in prevalence and intensity of infection. However, it has relatively little effect on transmission and regular re-treatment at intervals of one to three years is therefore usually necessary [1-4]. The intensity of re-infection after treatment in some cases may reach 50% of pre-treatment levels by about one year after treatment, especially in the younger age groups [5,6]. Even low-level recurring reinfection is likely to be associated with subtle but persistent morbidities such as anemia, undernutrition and diminished performance status [7]. One potential improvement to the strategy of regular chemotherapy may be to combine chemotherapy with mollusciciding of exposure sites. Such an approach has been successfully used in several studies. In St. Lucia in the West Indies, a community based chemotherapy programme resulted in a rapid reduction in prevalence and intensity of infection, and focal snail control delayed or prevented the expected resurgence of transmission [8]. A combination of chemotherapy and focal mollusciciding in Brazil reduced the prevalence of *S. mansoni* infection from between 12.5-40% to below 9% [9], while in the late 1950’s a combination of chemotherapy and mollusciciding helped to achieve the control of *S. mansoni* on Vieques Island in Puerto Rico [10]. In Burundi, however, focal snail control produced disappointing results [11]. This approach has not been attempted in Kenya, and has not been undertaken anywhere in comparison with chemotherapy alone at the same time and under similar environmental conditions.

This paper describes both operational and research aspects of introducing seasonal and focal mollusciciding with niclosamide (Bayluscide®) in a river in Makueni District, Kenya. A nearby community using a similar river and with intense transmission was selected for chemotherapy alone. Mollusciciding activities were undertaken to coincide with the natural fluctuations in snail numbers associated with rainfall patterns. The study was undertaken to elucidate the efficacy of mollusciciding after chemotherapy for long-term maintenance of low re-infection rates, and to develop a streamlined and simple procedure for use in Kenya and elsewhere endemic for schistosome infections.

## Methods

### Ethical clearance

This project was conducted as an integrated part of 4 other projects for which approval was obtained from the ethical committee at the Kenya Medical Research Institute, Nairobi, Kenya on August 11, 1992, January, 6, 1994, October 1, 1998 and July 7, 1999. For ethical reasons we decided to not return routinely collected *Biomphalaria pfeifferi* to the sites, either those with patent infection or those without because they might have prepatent infections.

### Study area

**Figure 1:**
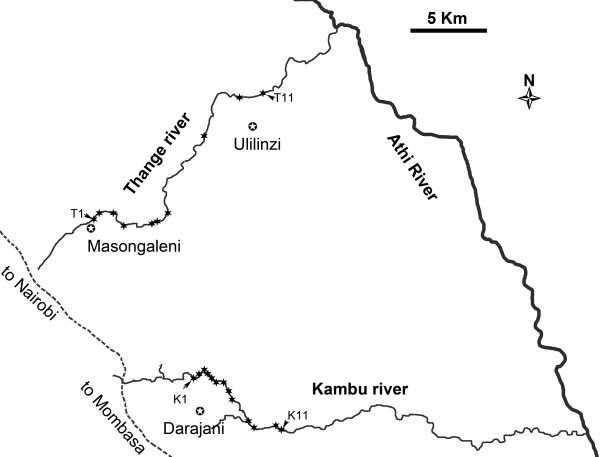
**The two study streams.** The stippled line indicates the Nairobi–Mombasa road and stars the sampling sites. Sites were numbered consequtively from T1 (S2° 28′ 24.96″; E38° 3′ 12.12″) to T11 (S2° 23′ 54.00″; E38° 9′ 20.64″) in the Thange stream (Non-intervention) and from K1 (S2° 34′ 1.17″ E38° 7′ 3.46″) to K11 (S2° 35′ 56.71″; E38° 9′ 47.14″) in the Kambu stream (Intervention). Ulilinzi and Darajani show the approximate position of schools in the two communities.

The general features of the study area have been described in previous publications [12,13]. The current study was carried out in the context of two rivers and their surrounding communities (Figure 1), i.e. the Kambu River, where community chemotherapy was followed by mollusciciding (Intervention), and the Thange River, which served as a control with no mollusciciding but chemotherapy of a local community only (Non-intervention).

Transmission of *Schistosoma mansoni* in Makueni District and in many other endemic areas in Kenya frequently occurs in streams, some of which may dry up during the dry season or be flushed by floods during heavy rains. There are two main annual transmission seasons in Makueni; these occur after the March/May long rains and November/December short rains [14].

The Kambu River passes close to Kambu Market on the Nairobi-Mombasa highway and close to Darajani Market some 5 km downstream. In an earlier study, examination for *S*. *mansoni* infection and associated morbidity at a nearby primary school (Nzoila Primary School) observed prevalence of infection among the school children of 90%, with 40% of these children displaying evidence of hepatosplenic disease [15]. Examination at other nearby primary schools in the Kambu area also indicated high prevalence of infection [12]. The source of infection in this area was Kambu River. Darajani village was selected for evaluation of *S. mansoni* transmission after mollusciciding.

Kambu River emerges about 7 km upstream from the study area as a series of springs originating from the nearby Kyulu Hills. The river, which on average is about 3 m wide and 0.2 m deep, flows through some rocky areas but mainly has a sandy bed often overlain with silt. Various types of emergent and floating vegetation suitable for snail proliferation are abundant. Kambu River is heavily used by humans for their domestic and personal activities and for watering their livestock from as far away as 10 km on either bank. Along its upper areas, where the present study was carried out, the river does not dry out completely during any time of the year.

Thange River was selected as the non-molluscicided control river, and Ulilinzi village, through which it flows, was used for comparative parasitological evaluation. The Thange River is similar in description to the Kambu, has its origins in the Kyulu Hills and flows in a parallel valley towards the Athi River (Figure 1). In both rivers transmission sites were numbered consecutively downstream from the source.

### Parasitology

A cohort of people was selected in both areas on the basis of the proximity of their households to the water contact sites (less than one kilometre from transmission sites). Ulilinzi was sparsely populated as opposed to the highly populated Darajani area. A baseline survey for *S. mansoni* infection was conducted in October 1996; 208 individuals aged 5 to 60 years from Ulilinzi, and 630 individuals aged 5 to 60 years from Darajani were examined. A stool sample was taken from each individual on three consecutive days, and from each sample two 50 mg thick smears were prepared using the Kato-Katz method [16]. The number of *S. mansoni* eggs per sample was counted. A few people who did not provide 3 samples were kept in the analyses. No systematic treatment with antischistosomal drugs had been given in the Thange area before these studies began but there had been annual treatments of children in some Kambu schools prior to 1996 and some of these were residents of the Darajani community. All infected people from the cohorts were treated with 40 mg praziquantel per kg body weight, while all other members of the two communities were offered free treatment using the same dosage. Neighbouring communities were not treated.

A follow up survey was conducted 5 weeks after treatment in both areas but it was found that in Darajani the initial treatment was less than satisfactory and all infected people were given a second treatment in January 1997 to reduce intensity of infection to a level comparable to that in Ulilinzi. The reason for the treatment failure most likely was that mainly children (up to teen age) did not swallow the tablets due to rumours about bad taste and severe side effects of the drug. In all subsequent treatments we were very careful to ensure that all swallowed the tablets. A 5 weeks follow-up survey was carried out after the second treatment in Darajani but this was based on only one stool sample and is therefore not included here. Another follow-up sampling was done in Darajani in May 1997 and this one was used for the analysis. Therefore, the follow up surveys were from December 1996 (5 weeks after treatment) at Ulilinzi and May 1997 at Darajani (about 4 months after the second treatment). Thereafter, annual surveys (Survey 1 – Survey 4) were conducted in each area, generally in November in Darajani (Intervention) and January the following year in Ulilinzi (Non-intervention). There was no other treatment for schistosomiasis infection in these areas except possibly by health centres. Praziquantel was available at the nearby Kibwezi health centre where infected individuals would be referred to. Most other clinics did not have praziquantel at that time.

### Snail sampling

Initially 11 sites on the Kambu and Thange rivers respectively (Figure 1), were purposively identified based on previous (unpublished) observations on extent of human water contact. During the pre-intervention all sites were sampled regularly but during the follow-up, only 9 sites on the Kambu river and 10 sites on the Thange river were sampled regularly. Each site was sampled for *B. pfeifferi* twice per month from January 1993 to December 2005. Sampling was carried out as described in [17], by one man searching each site for 15 minutes, using a standard flat, wire-mesh scoop (mesh size 2 mm). Snails were brought to a field laboratory in each area and examined for schistosome infection by cercarial shedding. *Biomphalaria pfeifferi* snails were placed individually in flat-bottomed glass vials (7.5 cm by 2.5 cm in height and diameter) containing clear filtered stream water and exposed to indirect sunlight for a maximum duration of 4 hours (900 h - 1300 h). The cercariae were categorized either as those of human schistosomes (*S. mansoni*) or other trematodes [18]. Snails were not returned to their sites after examination; the impact of this on snail population dynamics would probably be minor as snail density was high also in sections outside our sampling stations and drift of snails along the stream was significant (we did not quantify this).

### Rainfall

Rainfall was recorded daily at each field laboratory and presented as rainfall in mm per month.

### Mollusciciding

Bayluscide® WP 70% (niclosamide is the active molluscicidal ingredient) was used at the manufacturers recommended dose of 1 ppm for 8 hours [19]. The Kambu stream was treated at a concentration of 1 mg l^-1^ for 8 hours using a dispenser made of a 200-litre metal oil drum with a bottom siphoning mechanism (i.e. drip-feed application). At the same time, marginal water, small effluents and side ponds with no flow along the entire target stretch of the river, were treated with molluscicide applied with a 10-litre compression sprayer containing 50 g of molluscicide to give an estimated concentration of 2 mg l^-1^.

Mollusciciding began in August 1995, i.e. before the baseline parasitological survey, to ensure that the chosen methods were working. An attempt was made to target snails at the very source of the stream, which served as a refuge for snails, but this proved time consuming and too expensive in terms of chemical consumption. In February 1996, a routine for major, area-wide mollusciciding was adopted of one drip-feed application at a point about 2 km downstream from the main source (c. 3 km upstream from the first snail study-site) and a booster application at the middle of the study area, plus supplementary hand-spraying of marginal water along the target stretch of the river. This major mollusciciding was carried out twice a year, as snail populations began to recover from the preceding rainy seasons, in about February/March and August/September. In between these area-wide treatments, focal mollusciciding was carried out along the target stretch of the river with backpack sprayers in and around sites where snails were found.

Initially, flow was estimated by means of a V-notch weir but later we estimated average water flow using a floating wine bottle cork, with satisfactory results. The cork was dropped at point 0 and the time it took to reach the 10-m mark taken. The average width and depth of the 10-metre section were also taken. The flow rate was then calculated as shown in [19].

The efficacy of the molluscicide was estimated by exposing caged sentinel snails downstream at various points. Control snails were also placed upstream in areas where the molluscicide was not introduced. The snails were introduced in 10 × 10 × 10 cm cubic cages surrounded with plastic mosquito netting that was not large enough to allow for snails to crawl out. Ten snails were placed in each cage, and one cage placed at intervals of one km.

### Training

Five persons from the local community were trained in mollusciciding, evaluation of snail population sizes and identification of infected snails.

### Analysis

Infection status at baseline (infected/not infected) and egg counts were compared between the two areas after adjusting for age class and gender using generalized linear models, i.e. logistic regression using a logit link function for infection status and negative binomial regression using a log link function for egg counts. Egg counts from the three (for some samples less) consecutive samples were summed and the total amount of faeces actually examined was used as an offset in these analyses. The ancillary parameter (α) in the variance function (μ + αμ^2^) was estimated using full maximum likelihood estimation as described by [20]. Model fit was checked by assessing over-dispersion using the chi-square based dispersion statistics and standardized deviance residuals were checked to identify potential outliers [20].

For comparison between areas of pre-intervention snail counts (monthly counts in sites for two years i.e. 1993 and 1994), population averaged generalized estimating equations [21] were used. For infections in snails a logit link was used. Different correlation structures were tried and the one chosen was based on the QIC statistic [21]. The best was an autoregressive correlation structure with a lag of 1.

Since infections in people differed between the two areas at baseline, the evaluation of treatment (mollusciciding) effects would need to adjust for this difference. Data were arranged as panel data (a panel is a person or a snail sampling site) where each panel was examined once a year. For infections in people, generalized estimating equations on the yearly survey data adjusting for baseline infection status or intensity of infection (eggs per g faeces) using logistic regression or negative binomial regression in population averaged generalized estimating equations were used. For snail data, the total number of snails sampled per site was calculated for each year and comparison of treatment effect was adjusted for snail counts during the two pre-treatment years. Incidence rate ratios (relative risk) between the two areas were calculated between successive surveys after adjusting for age group, gender and baseline infection status if significant and interval between surveys was entered as the exposure using a generalized linear model with binomial distribution and a log-link function. P-values below 0.05 were taken to indicate significant differences.

## Results

### Snails –pre-intervention

**Figure 2:**
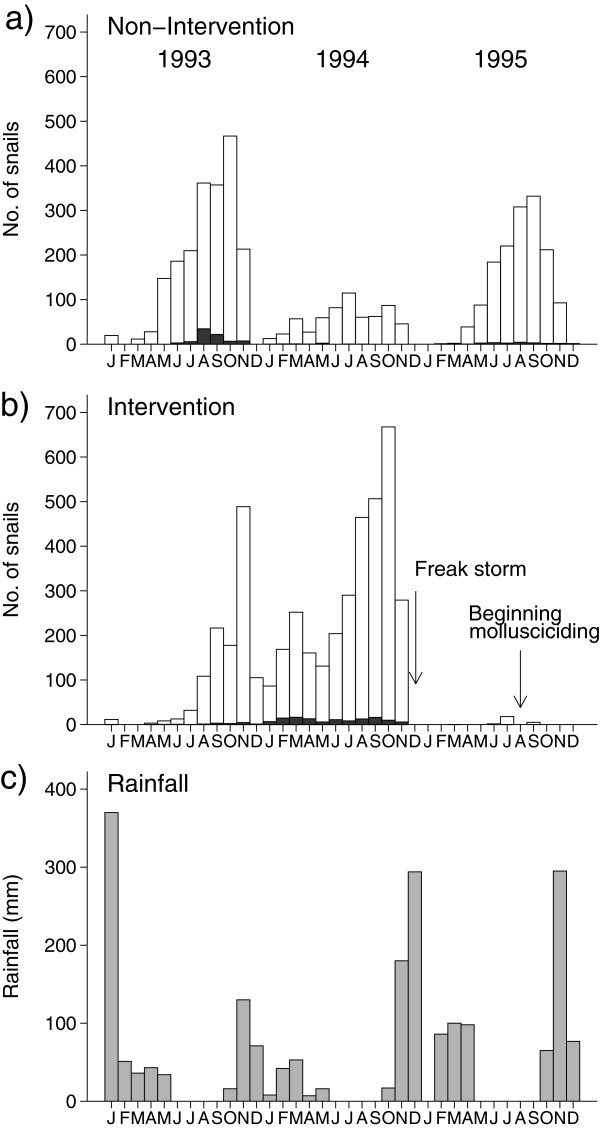
**Numbers of *Biomphalaria pfeifferi *(open columns) and *Schistosoma mansoni *infected snails (black columns) per month per site in two streams, Thange a) and Kambu b), during the pre-intervention years (1993 and 1994) and the first year of mollusciciding, together with rainfall c).**

Pre-intervention snail sampling in 1993 and 1994 is shown in Figure 2. Rainfall followed the expected pattern of a short period with high rainfall in November/December of each year, and a longer period of lower rainfall between March and May. Density of *B. pfeifferi* increased from about May to November when density declined markedly. These trends, however, were not the same in both years and thus a significant interaction was found between season and year (p < 0.001) and between year and area (p < 0.001). Also for the number of *S. mansoni* infected snails interactions between season and year (p < 0.001) and between year and area (p < 0.001) were significant.

**Figure 3:**
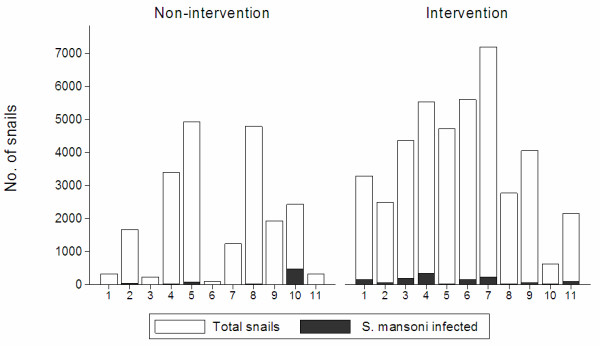
**Total number of *Biomphalaria pfeifferi *collected in each site (Figure**1**) during the two pre-intervention years, 1993 and 1994.** A few of these sites were excluded from the follow-up analysis.

There was considerable variation in both the number of snails collected and the percentage of snails that shed *S. mansoni* cercariae within and between sites along both rivers (Figure 3). Sites 1–3 in the non-intervention area and site 10 in the intervention area were not sampled regularly during the follow-up period and were therefore excluded from the following analysis. The highest recorded number of snails at any site visit along the non-intervention river was 992 in 1993 and along the intervention stream it was 1024 at one site in 1994. In the non-intervention river, sites 4, 5 and 8 had the highest snail counts and jointly these sites accounted for 68.6% of all snails found in this area during the two pre-intervention years. In the intervention area, there was less variation among sites; lowest counts were found in sites 2 and 11 and highest counts in sites 4, 6 and 7. The latter three sites accounted for 43.5% of all snails found during the pre-intervention years.

The odds of finding *S. mansoni* infections in snails in the non-intervention area were higher in sites 5, 10 and 11 than in the other sites combined (p < 0.001) and infections were more commonly found in 1993 than in 1994. Infected snails were found in all sites, but site 10 accounted for 77.0% of all infected snails in the non-intervention area during the pre-intervention period and sites 4, 5 and 10 accounted for 91.5% of all infected snails. In the intervention area, the odds of finding infections in snails was lower in sites 5 and 8 than in the other sites but differences among the other sites were also significant (p < 0.001). Especially, sites 1, 3 and 4 had high odds of infections. Infections were more commonly found in 1994 than in 1993 (p < 0.001). Also in the intervention area, infected snails were found in all sites and variation between sites was less than in the non-intervention area. Thus sites 3, 4 and 7 accounted for 57.8% of all infected snails during the pre-intervention period.

A freak storm during the short rainy season from November to December 1994 washed away *B. pfeifferi* populations and caused adverse alteration of snail habitats in the Kambu River by washing out vegetation and depositing large amounts of silt; the storm did not affect the Thange River. As a consequence, and in addition to the effects of the rains that fell between February and Apri11995, no snails were found in the Kambu sites until June 1995 when a small number of snails were found.

### Mollusciciding

The first mollusciciding in August 1995 consumed a total of 6.1 kg of Bayluscide, while subsequent routine applications consumed 10 kg of the molluscicide per treatment (i.e. 2 per year), including the supplementary hand spraying. In between these area-wide treatments, focal mollusciciding was carried out with backpack sprayers in and around sites where snails were found, using an average of 3 kg per year. Caged snails were taken to Darajani field laboratory and placed in clean stream water for 24 hours for recovery. All caged snails exposed in the molluscicided section were found dead up to a distance of 10 kilometres downstream, but those exposed 13 km downstream survived. There was no mortality in the snails exposed upstream in the untreated section of the stream.

### Snails –during intervention

**Figure 4:**
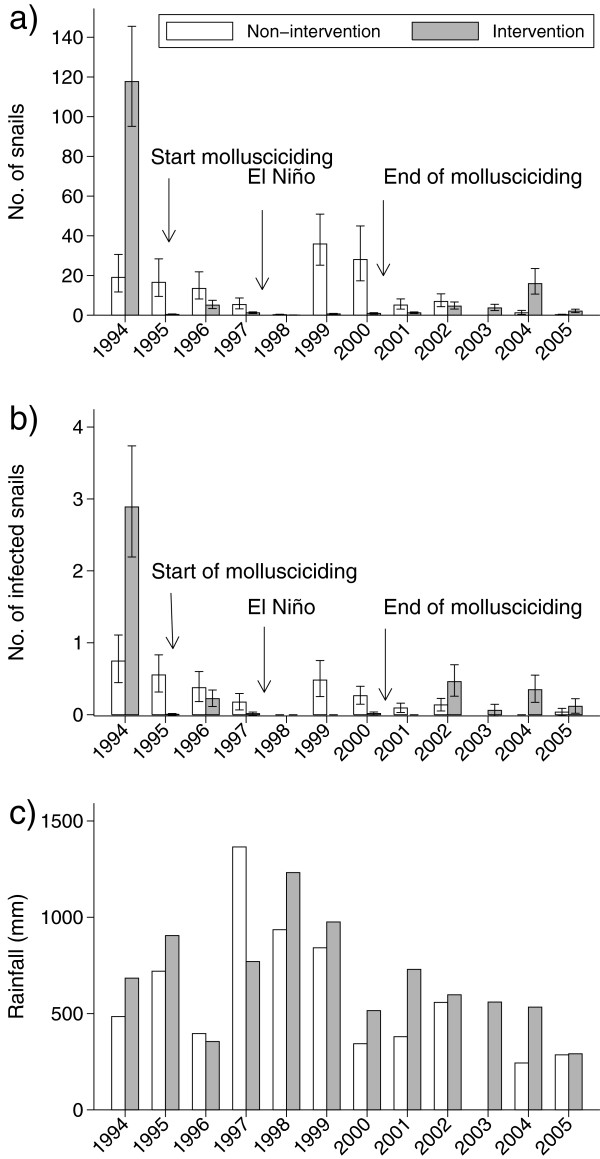
**Mean total number of *Biomphalaria pfeifferi *(a) and number of infected *B. pfeifferi *(b) collected per site per year during 1994 to 2005, together with rainfall (c).** Error bars represents 95% CL.

The climatic phenomenon El Nino occurred in 1997, and this was associated with a disruption of normal rainfall patterns in both study areas. Abnormally high rainfall was measured during the period November 1997- January 1998, and the rains did not completely abate until June 1998. Prior to El Nino, snail populations at sites along the non-intervention river typically crashed with the onset of the November rains, and recovered after the March-May rains. During 1997 and 1998 there was a population crash that lasted from November to November of the following year, after which the previously observed fluctuations in snail populations was resumed. Population averaged modelling with a autoregressive 1 correlation structure showed that baseline snail count was a significant predictor of follow-up counts (p < 0.001). Rainfall was a significant predictor and a 100 mm increase was associated with a reduction in snail counts by 27% (p < 0.001). An increase in the baseline count of 100 snails was associated with a 43% increase in follow-up counts. The interaction between baseline count and area was not significant and was excluded from the final model. Area (Intervention/non-intervention) was a significant predictor (p < 0.001) of follow-up snail count and so was year (p < 0.001), and the interaction between these two predictors was significant (p < 0.001). Thus counts in the intervention area was 1.5, 25.1, 6.4, 6.7, 1.2 and 1.6% of that in the non-intervention area during years 1995, 1996 1997, 1998, 1999 and 2000, respectively when adjusting for baseline snail counts and rainfall. Mollusciciding was terminated at the end of 2000 and during subsequent years snail density in the intervention stream increased although not to the same level as pre-intervention (Figure 4).

### Parasitological observations at baseline

**Figure 5:**
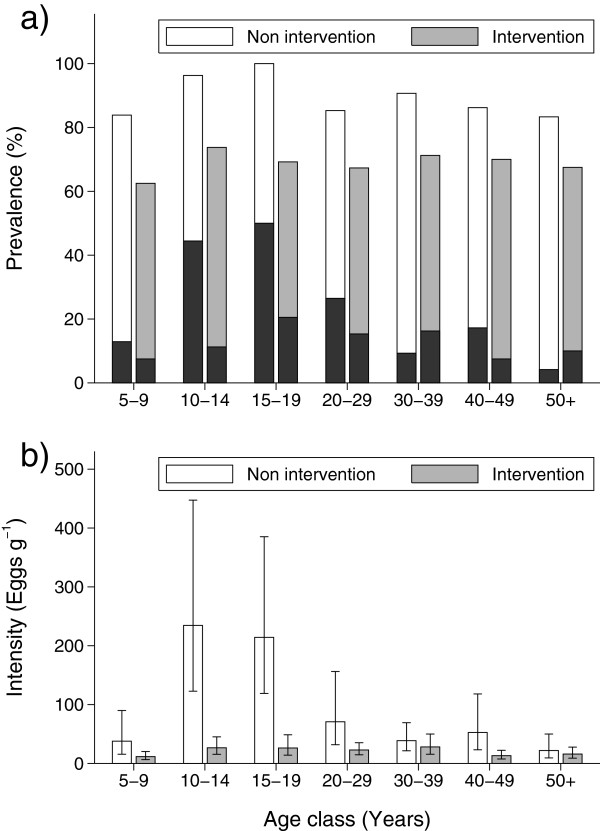
**Prevalence (a) of *Schistosoma mansoni *infection and heavy infections (black bars) and intensity of infection (b) by age group in the intervention and non-intervention area at base line.** Error bars represents 95% CL.

Odds of *S. mansoni* infection at baseline among people in the intervention area were lower than that in the non-intervention area (OR = 0.27; p < 0.001) when adjusted for the effects of age group and gender, i.e. males had higher odds of infection than females (OR = 1.87; p < 0.001). Age group was not significant but was retained in the model. Similarly, odds of heavy infection (epg > =400) were lower in the intervention than in the non-intervention area (OR = 0.16; p < 0.001), when adjusted for the effects of age group and sex. Effect of gender was not significant while age group was significant (p < 0.001). There was, however, a significant interaction between age group and area (p < 0.05); especially school aged children who had higher prevalence of heavy infection in the non-intervention area than in the intervention area (Figure 5a).

Egg counts modelled as a Poisson distribution were significantly over-dispersed, wherefore we used negative binomial regression. The ancillary parameter was estimated to be 3.4231 and a model including area, gender, age group and the interaction between area and gender showed no signs of over-dispersion (dispersion statistics = 1.01). Egg counts were lower in the intervention area than in the non-intervention area (p < 0.01), males had on average 36% higher egg counts than females, and age group was a significant predictor (<0.01) as well. There was, however, a significant interaction between area and age group (p < 0.05).

### Parasitological observations during follow-up

In the follow-up survey after treatment and the following 3 annual surveys a cumulative total of 261 infections were recorded in the non-intervention area and 224 in the intervention area. Of these, 78 (29.9%) and 73 (32.6%) in the non-intervention and intervention area, respectively were negative in the survey that followed the one where they were detected indicating that some people were treated, presumably at health centers although this could also be a random variation in sensitivity at low egg counts. A total of 23 (88%) and 29 (73%) of the people found positive during the follow-up survey after treatment were also positive in the last survey in the non-intervention and intervention area respectively and of these 8 and 15, respectively, were found positive during all surveys. The maximum observed egg count during the various follow-up surveys was 557 and 750 eggs g^-1^ in the non-intervention and intervention area, respectively (Table  1). Maximum egg-counts for people who were also positive during the preceding survey were higher but these high intensities of infection were found in only a few people.

**Table 1:** Number of people found infected, maximum intensity of infection and geometric mean intensity of infection in the non-intervention and intervention areas in Makueni District, Kenya

	**Number of cases**		**Maximum intensity (eggs g**^**-1**^**)**		**Geometric mean intensity (eggs g**^**-1**^**) (95% CL)**	
	**Non-intervention**	**Intervention**	**Non-intervention**	**Intervention**	**Non-intervention**	**Intervention**
**Baseline survey (October 1996)**						
Negative	22	194				
Positive	154	380	1440	3340	90 (70–117)	77 (65–92)
Positive also at follow-up	32	56	1403	1303	179 (101–318)	99 (68–145)
Follow-up survey*						
New cases	1	5	420	25	420	11 (4–26)
Positive pre-treatment	32	56	300	1000	29 (20–42)	20 (13–29)
**Survey 1 (1997/1998)**						
New cases	63	43	470	750	20 (14–30)	11 (6–18)
Positive at follow-up	24	31	407	1103	24 (13–45)	21 (12–38)
**Survey2 (1998/1999)**						
New cases	18	32	397	525	8 (4–14)	11 (7–17)
Positive at survey 1	36	35	1333	655	8 (5–13)	20 (11–34)
**Survey 3 (1999/2000)**						
New cases	94	36	557	303	22 (17–28)	9 (6–15)
Positive at survey 2	45	40	1380	417	26 (16–42)	21 (13–32)
**Survey 4 (2000/2001)**						
New cases	17	47	523	743	15 (7–32)	9 (7–13)
Positive at survey 3	78	45	2527	833	48 (32–72)	29 (17–47)

*) treatment was given in October 1996 and this survey was conducted 5 weeks post-treatment in the non-intervention area, while a second treatment was given in January 1997 in the intervention area and the survey data presented here are from 4 months post-treatment.

**Figure 6:**
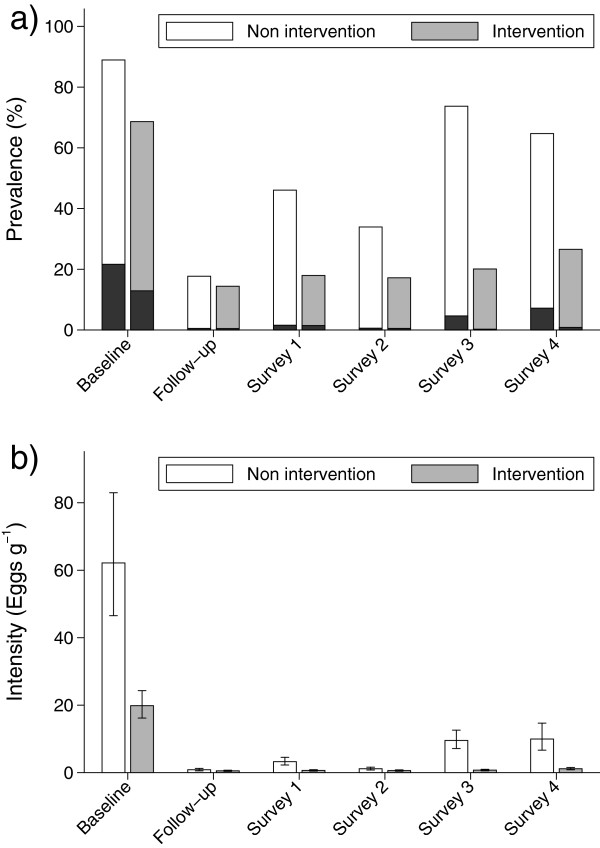
**Prevalence (a) of *Schistosoma mansoni *infection and heavy infections (black bars) and intensity of infection (b) as geometric mean by year in the intervention and non-intervention areas.** Error bars represents 95% CL. The baseline was from October 1996, while surveys 1–4 were from November in the Intervention area and the following January in the Non-intervention area during the years 1997/1998, 1998/1999, 1999/2000 and 2000/2001, respectively.

Prevalence of infection and heavy infection recorded at each survey in each community is shown in Figure 6. Since prevalence of infection differed between areas at the baseline survey, adjustment of follow-up effects was made for infection status of each person at baseline. In a GEE model, the odds of infection during follow-up was higher among those who were positive at baseline than among those who were originally negative (OR = 2.84, p < 0.001). There was a significant effect of age group (p < 0.001) and of area (OR = 0.20; p < 0.001), while there was no interaction between age group and area and this interaction term was excluded from the final model. Males had higher odds of infection than females (OR = 1.44, p < 0.01). Year was a significant predictor (p < 0.001) but there was a significant interaction between year and area. Thus the odds of infection in the intervention area were 0.65, 0.24, 0.41, 0.08 and 0.20 of that in the non-intervention area during the follow-up survey and the 4 subsequent annual surveys, respectively.

Incidence ratio between areas adjusted for age group and gender at the follow-up survey after chemotherapy was not significant, while during the following 4 annual surveys these ratios (95% CL) were 0.58 (0.39-0.85), 0.33 (0.18-0.60), 0.14 (0.09-0.21) and 0.45 (0.26-0.75), respectively. Few heavy infections were observed during the post-treatment surveys, 25 in the non-intervention area (20 of these during 2000) and 14 in the intervention area.

The effect of treatment on egg counts was more marked. The first treatment achieved a reduction of over 95%, rising to 99.15% after the supplementary treatment given 3 months later. Intensity of infection at baseline was a significant predictor of follow up egg count (p < 0.001) when adjusting for gender, age group, year, interaction between area and age group and between area and year; a 100 eggs increase in baseline epg was associated with a 9.8% increase in follow-up egg count. Males had higher egg counts than females (count ratio: 3.08, p < 0.001). Also age group (p < 0.001) and year (p < 0.001) were significant predictors, but both had significant interaction with area (p < 0.001 for both terms). Ratios between egg count in intervention and non-intervention area were 0.43, 0.17, 0.45, 0.13 and 0.18 during the follow-up and the 4 annual surveys, respectively. Thus egg counts were lower in the intervention area than in the non-intervention area during all years of mollusciciding when adjusted for baseline differences.

## Discussion

Schistosomiasis continues to exert a burden on public health in many communities of the tropics and sub-tropics even where National Control Programmes operate. This is due to the fact that chemotherapy alone does not prevent re-infection. We hypothesized at the outset of this project that mollusciciding, using local knowledge of snail breeding sites, could lead to an additional benefit to the populations at risk of infection. Mollusciciding is no longer a feature of many schistosomiasis control programmes but our results press home the fact that additional measures against the snail population can have measurable benefits. The major observations of this study are that the snail numbers were reduced significantly at all sites along the Kambu River (Intervention area), and that both prevalence and intensity of infection after chemotherapy remained lower in the Darajani community, close to Kambu river, than in the non-intervention area, Ulilinzi village, close to Thange River during the follow-up period. The combination of chemotherapy followed by intermittent mollusciciding, therefore, had a significant protective effect not only for the Darajani community but also for neighbouring communities also relying on the Kambu River for their water requirements, i.e. an estimated population of over 30,000 [22].

In an area where environmental conditions favour the proliferation of intermediate host snails, *Biomphalaria pfeifferi*, complete elimination of snails may be impossible to achieve, especially in stream habitats. Further, after mollusciciding some snails may be found at some habitats, especially where suitable ponds or pools form. Formation of such pools is usually caused by fast currents during rains that wash away sand at some points along the riverbed, leaving deep furrows. The location of such pools may change from one rainy season to the next. It is therefore necessary to identify such sites and check for snails regularly. This is in marked contrast to a study in the Gambia, where mollusciciding of seasonal rainwater pools progressively reduced snail population density and markedly reduced transmission of *S. haematobium*[23].

Where snails occur in streams, as in the present study area, rainfall plays an important role in reducing snail populations and the population “starts afresh” after each rainy season, but the effect is only temporary as re-population occurs rapidly. This is partly because close to the source of such streams several “snail pockets” may exist and some of them may be of very small size and yet support a thriving snail population. It may not be possible to locate and treat all these reservoirs of snails. Thus after mollusciciding is stopped snail populations will soon recover. This study has shown that taking advantage of rainfall by timed mollusciciding helps to keep snail populations low for a prolonged period. It may, however, not be straightforward to estimate when rains may cause depletion of snails, since during this study the rainfall did not follow the same pattern from year to year. During the March-May long rainy seasons the rains were generally low. The El Niño rains from November 1997 to January 1998, in contrast, were devastating for snail habitats in both Kambu and Thange rivers, resulting in depletion of monthly snail recoveries in 1998 until August for Kambu and September for Thange.

Focal mollusciciding using the compression sprayers hardly caused any noticeable effect on non-target organisms, especially fish, but area-wide mollusciciding did kill some fish (data not included). Kambu River is not an important area for fishing nor does the community rely on fish as a source of food, but it joins a major river, the Athi, which is an important source of fish. Due to dilution, the molluscicide effect was not felt in the Athi River during the area wide mollusciciding (the late Dr RF Sturrock, personal communication).

Several important questions are associated with the use of molluscicide to prevent reinfection rather than relying on repeated treatment of exposed individuals to maintain low reinfection levels. The cost effectiveness of the mollusciciding approach is particularly important. In the present study, the cost of a single round of chemotherapy of Darajani community was approximately 1.8 times higher than the annual mollusciciding cost (data not shown), but would obviously be different at present due to the reduction in praziquantel cost. Had all persons at risk been treated annually, the relative cost would have been considerably higher, and such an approach would not have prevented re-infection [2,7]. It is also important to ask whether or not intermittent mollusciciding can have a demonstrable effect on morbidity. In this context, it has been shown that the mollusciciding of the River Kambu was associated with both a lack of reinfection amongst a cohort of school children from the nearby Mbeetwani community, and also a regression of hepatosplenomegaly over a three year period [24,25]. A lack of re-infection amongst the Mbeetwani cohort led to observations concerning the effects of malaria exposure on hepatosplenic disease associated with schistosomiasis [26,27]. Taken together, these two sets of studies indicate that whilst reduction in infection is clearly demonstrable through a combination of mollusciding and chemotherapy, any causally related effect on morbidity is likely to be a function of various factors including the presence of co-infections of different species. Defining the nature of this ‘function’ will require a mixed-methods approach in future studies to ensure that a more complete understanding of schistosome ecology is available to inform the development of optimised control programmes. With some countries shifting schistosomiasis control strategy from morbidity control to elimination [28,29], transmission control becomes important and mollusciciding could become essential to achieve this and there is need to develop new molluscicide formulations or new strategies of application adapted to the local conditions [29,30].

## Conclusion

This study has demonstrated that chemotherapy followed by intermittent mollusciciding can have a significantly higher impact on re-infection than a single round of chemotherapy, and may be more cost-effective than repeated mass treatments of the human beings. These results were obtained in a specific Kenyan setting, but nonetheless demonstrate that a combined approach to schistosome control is feasible and has demonstrable benefits in terms of reducing infection and morbidity in communities where transmission is high.

## Competing interests

The authors declared that they have no competing interests.

## Authors’ contributions

HCK, HM and JHO formulated the original project idea, but all authors have been involved in the design and later adjustments over the years to ensure coordination with other separate projects in the area. HCK was overall responsible for the data collection and coordination. HM and MB took the lead in analysing the data. All authors read and approved the final version of the manuscript.
